# CRISPR-Cas3-based diagnostics for SARS-CoV-2 and influenza virus

**DOI:** 10.1016/j.isci.2022.103830

**Published:** 2022-01-30

**Authors:** Kazuto Yoshimi, Kohei Takeshita, Seiya Yamayoshi, Satomi Shibumura, Yuko Yamauchi, Masaki Yamamoto, Hiroshi Yotsuyanagi, Yoshihiro Kawaoka, Tomoji Mashimo

**Affiliations:** 1 Division of Animal Genetics, Laboratory Animal Research Center, Institute of Medical Science, The University of Tokyo, Tokyo 108-8639, Japan; 2 Division of Genome Engineering, Center for Experimental Medicine and Systems Biology, Institute of Medical Science, The University of Tokyo, Tokyo 108-8639, Japan; 3 Advanced Photon Technology Division, RIKEN SPring-8 Center, Hyogo 679-5148, Japan; 4 Division of Virology, Department of Microbiology and Immunology, Institute of Medical Science, The University of Tokyo, Tokyo 108-8639, Japan; 5 C4U Corporation, Osaka 565-0871, Japan; 6 Division of Infectious Diseases and Applied Immunology, Institute of Medical Science, The University of Tokyo, Tokyo 108-8639, Japan; 7 Department of Pathobiological Sciences, School of Veterinary Medicine, University of Wisconsin-Madison, Madison, WI 53711, USA

**Keywords:** Diagnostics, Virology

## Abstract

CRISPR-based diagnostics (CRISPR-dx), including the Cas12-based DETECTR and Cas13-based SHERLOCK Class 2 CRISPRs, have been used to detect the presence of DNA or RNA from pathogens, such as the 2009 pandemic influenza virus A (IAV) and the 2019 novel coronavirus SARS-CoV-2. Here, we describe the collateral single-stranded DNA cleavage with Class 1 type I CRISPR-Cas3 and highlight its potential for development as a Cas3-mediated rapid (within 40 min), low-cost, instrument-free detection method for SARS-CoV-2. This assay, which we call Cas3-Operated Nucleic Acid detectioN (CONAN), not only detects SARS-CoV-2 in clinical samples, but also offers specific detection of single-base-pair mutations in IAV variants. This tool allows rapid and accurate point-of-care testing for patients with suspected SARS-CoV-2 or drug-resistant IAV infections in hospitals.

## Highlights

• Type I CRISPR-Cas3 shows *trans*-cleavage activity on nearby nonspecific ssDNA
• We developed CONAN as a method to easily and accurately detect viral RNA
• CONAN provides rapid, low-cost, and instrument-free detection of SARS-CoV-2 and IAV
• CONAN also allows the specific detection of single-base-pair mutations

## Graphical abstract

**Figure:**
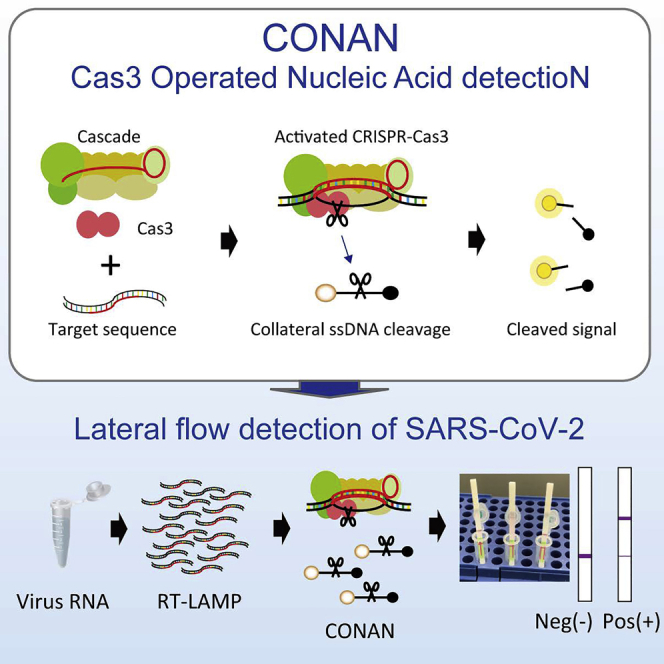

## Introduction

Over the last 20 years the world has faced several epidemics and pandemics that have seriously threatened global public health, including the 2002 severe acute respiratory syndrome (SARS), the 2009 influenza virus A (IAV) pandemic, the 2012 Middle East respiratory syndrome (MERS), the 2014 Ebola outbreak, and the 2019 coronavirus disease (COVID-19) pandemic. This history highlights the urgent necessity for fast, sensitive, and specific diagnostic tools for virus surveillance, including antimicrobial resistant and emerging virus variants. Many countries use assays based on real-time reverse-transcriptase PCR (RT-qPCR) to detect viruses. However, the results of such assays on clinical samples from people with suspected virus infections are generally not ready until the day after sample collection because the samples need to be shipped to reference laboratories for accurate diagnostic testing (Bustin and Mueller, 2005; Corman et al., 2020; Ravina et al., 2020). RT-qPCR assays also require expensive equipment and well-trained personnel for their operation. Alternatively, rapid antigen tests can directly detect viral components without the amplification steps needed for RT-PCR; however, the evidence for their sensitivity and diagnostic accuracy require evaluation (Green and StGeorge, 2018). In contrast, serology antibody tests with lateral flow immunoassays can rapidly and sensitively determine the infection rate in a population, although IgG antibodies to viruses are generally only detectable 10–14 days postinfection (Kumar and Henrickson, 2012; Theel et al., 2020).

To overcome such limitations in the current diagnostic technologies, CRISPR-based diagnostics (CRISPR-dx) have been used to rapidly, robustly, and sensitively detect emerging viruses. These systems rely on type V CRISPR-Cas12, otherwise called DETECTR (**D**NA **E**ndonuclease-**T**arg**E**ted **C**RISPR **T**rans **R**eporter) (Chen et al., 2018) or type VI CRISPR-Cas13, otherwise called SHERLOCK (**S**pecific **H**igh-sensitive **E**nzymatic **R**eporter Un**LOCK**ing) (Gootenberg et al., 2017). Both of these Cas enzymes, but not Cas9, exhibit nonspecific endonuclease activity in *trans* after binding to a specific cis target via programmable CRISPR RNAs (crRNAs). By combining isothermal amplification methods (e.g., **R**ecombinase **P**olymerase **A**mplification, RPA (Piepenburg et al., 2006), or **L**oop-mediated isothermal **AMP**lification (LAMP) (Notomi et al., 2000) with reporting formats such as lateral flow detection with antigen-labeled reporters (Kellner et al., 2019), DETECTR (Broughton et al., 2020; Ding et al., 2020; Lucia et al., 2020) and SHERLOCK (Ackerman et al., 2020; Metsky et al., 2020) have recently been used for rapid and highly sensitive SARS-CoV-2 detection. Furthermore, during the peer reviewing period of this manuscript, type III CRISPR-Cas systems have also been reported for SARS-CoV-2 diagnosis (Santiago-Frangos et al., 2021). The Cas13-based strategy, PAC-MAN (**P**rophylactic **A**ntiviral **C**RISPR in hu**MAN** cells), has also been shown to inhibit and degrade SARS-CoV-2 viral RNA in respiratory epithelial cells (Abbott et al., 2020).

**Figure 1:**
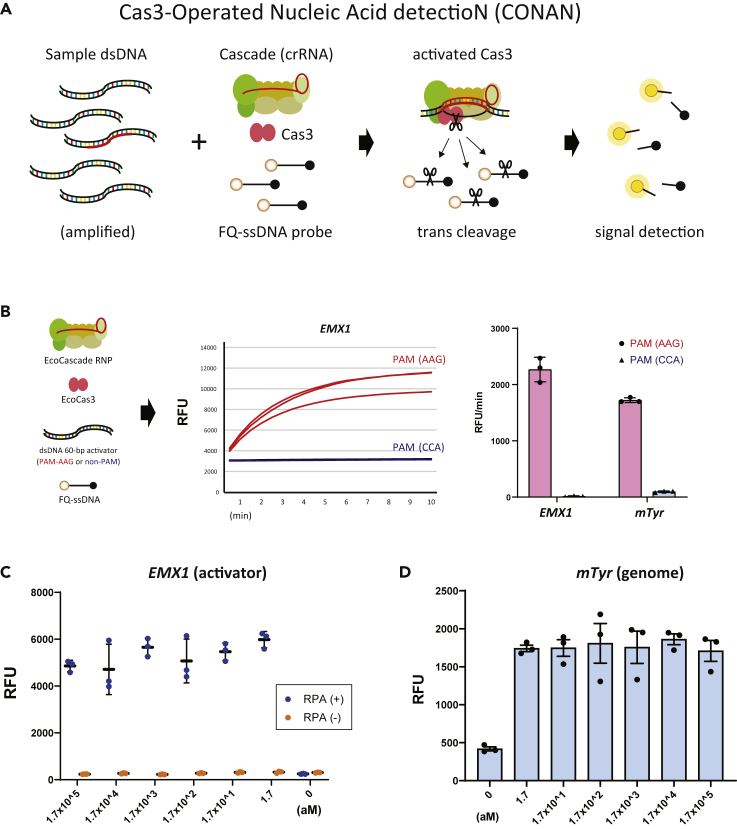
Cas3-operated nucleic acid detection (CONAN) (A) Schematic representation of the CONAN *in vitro* nucleic acid-detection platform. The *E. coli* CRISPR-Cas3 complex contains Cas3, Cas5, Cas6, Cas7, Cas8, and Cas11 proteins and CRISPR RNA (crRNA), FQ-ssDNA, fluorophore, and quencher-labeled single-stranded DNA probe. (B) Collateral ssDNA cleavage activity measured by incubation of EcoCas3-EcoCascade/crRNA complex with a 60-bp dsDNA activator containing a target sequence flanked by a PAM and an FQ-labeled ssDNA probe in reaction buffer containing MgCl_2_, CoCl_2_, and ATP for 10 min at 37 °C. CRISPR-Cas3 mediated collateral ssDNA cleavage after targeting *hEMX1*-dsDNA in fragments with PAM (AAG, red), but not in fragments with non-PAM (CCA, blue), quantitatively represented by relative fluorescent units (RFU) per min; increasing rate of RFU/min (right). Means (n = 3), and standard deviations. (C) CONAN assay on isothermal RPA amplicon products (blue) detected a single copy of the *EMX1* activator fragments (1.7 a.m.); RFU at 10 min. Means (n = 3), and standard deviations. (D) CONAN RPA also detected a single-copy *mTyr* activator (1.7 a.m.) when mixed with mouse genomic DNA. Means (n = 3), and standard deviations.

Class 2 CRISPRs (type V Cas12, type VI Cas13, and type II Cas9) only use a single Cas protein, whereas the three Class 1 CRISPRs (I, III, and IV) use multiple different Cas proteins. Several studies have reported on the mechanisms involved in type I CRISPR interference (Guo et al., 2017; Hochstrasser et al., 2014; Loeff et al., 2018; Mulepati and Bailey, 2013; Redding et al., 2015; Rutkauskas et al., 2015; van Erp et al., 2018; Westra et al., 2012; Xue et al., 2017). Type I CRISPR-Cas complexes have been reported to be seahorse-like structures containing Cas5, Cas6, multiple Cas7, Cas8 (Cse1), and two Cas11 (Cse2), and are named Cascade (Chowdhury et al., 2017; Hayes et al., 2016; Jackson et al., 2014; Mulepati et al., 2014; Sashital et al., 2012; Wiedenheft et al., 2011; Xiao et al., 2017). Cas3, a protein containing a serine-phenylalanine (SF) 2-helicase domain and a histidine-aspartic (HD) acid-nuclease domain, degrades its target DNA in a unidirectional ATP-dependent manner (Sinkunas et al., 2011; van Erp et al., 2018; Westra et al., 2012; Xiao et al., 2017, 2018). Interestingly, *Streptococcus thermophiles* Cas3 (Sinkunas et al., 2011) and *Methanocaldococcus jannaschii* Cas3 enzymes (Beloglazova et al., 2011) have an indiscriminate type of single-stranded DNA (ssDNA) cleavage when activated by Mg^2+^ bound to the catalytic site of the HD domain. In contrast, activation of the HD domain in *Escherichia coli* Cas3 (EcoCas3) (Mulepati and Bailey, 2013; Yoshimi et al., 2021) and *Thermus thermophilus* Cas3 (Mulepati and Bailey, 2011) is elicited by transition metal ions such as Co^2+^ and Ni^2+^, but not by Ca^2+^ and Mg^2+^. Despite type I-E *E. coli* CRISPR being one of the most thoroughly biochemically characterized *in vitro* plasmid DNA degradation-inducing systems (Hidalgo-Cantabrana and Barrangou, 2020; Zheng et al., 2020), whether the CRISPR-Cas3 system can mediate the target-activated, nonspecific ssDNA cleavage reported for Cas12 and Cas13 (Chen et al., 2018; Gootenberg et al., 2017) remains an open question. Here, we report on the third CRISPR-dx platform, **C**as3-**O**perated **N**ucleic **A**cid detectio**N** (CONAN) (Figure 1A). When combined with isothermal amplification methods, CONAN provides a rapid, sensitive, and instrument-free detection system for SARS-CoV-2 point-of-care test (POCT) applications.

## Results

### *Trans*-cleavage activity of the type I CRISPR-Cas3 system

Owing to the poor solubility and easy aggregation of EcoCas3 protein at 37 °C (Beloglazova et al., 2011; Mulepati and Bailey, 2013; Sinkunas et al., 2011; Westra et al., 2012), we purified it using the baculovirus expression system (Hitchman et al., 2009) with Sf9 insect cells cultured at 20°C (Figure S1 and STAR Methods). EcoCas3 did not show nonspecific ssDNA cleavage activity in Mg^2+^-containing buffer, consistent with previous results (Mulepati and Bailey, 2013), whereas *Thermobifida fusca* Cas3 (TfuCas3) (Dolan et al., 2019) did show indiscriminate cleavage activity (Figure S2). We next co-expressed and size exclusion chromatographically (SEC)-purified the core complex of *E. coli* Cascade (EcoCascade) proteins and crRNA from *E. coli* JM109 at 37°C (Figure S1 and STAR Methods). To investigate whether the type I CRISPR-Cas3 system carries its *trans*-cleavage activity on nearby nonspecific ssDNAs in a similar fashion as type V Cas12 and type VI Cas13, we used the collateral cleavage assay previously reported for DETECTR (Chen et al., 2018) and SHERLOCK (Gootenberg et al., 2017). Briefly, we assembled the EcoCas3/EcoCascade-crRNA complex, a 60-bp double-stranded DNA (dsDNA) actftor for the specific target gene, human *EMX1* (*hEMX1*) or mouse *Tyr* (*mTyr*) (Tables S1 and S2), and a fluorophore quencher (FQ)-labeled ssDNA probe in the Mg^2+^ reaction buffer (Chen et al., 2018; Gootenberg et al., 2017; Mulepati and Bailey, 2013). After 10 min incubation at 37 °C, we observed nonspecific *trans*-ssDNA cleavage from the EcoCas3/EcoCascade after target-specific dsDNA cleavage, which recognized the protospacer adjacent motif (PAM) (AAG), but not the non-PAM (CCA) (Figure 1B).

We next performed several additional experiments to understand the biochemical activities necessary for collateral cleavage activity by the Cascade-Cas3 system. First, the longer 3-Kb dsDNA activator (*hEMX1* plasmid), instead of the 60-bp short fragments, similarly mediated the *trans*-ssDNA cleavage by EcoCas3/EcoCascade (Figure S3). Second, changes in the concentration of the FQ-ssDNA probe clearly correlated with the signal intensity (Figure S4), suggesting that Cas3 can cleave several copies of ssDNA per bound Cascade. Third, in ATP-free reaction buffer (−), the collateral activity of the EcoCas3 protein was similar to that of wild-type EcoCas3 and the SF2 motif III S483A/T485A (dead helicase mutant, dhCas3) mutant (Morisaka et al., 2019) in ATP (+) buffer, meaning that the helicase activity of EcoCas3 is not essential for collateral cleavage (Figure S5). Finally, omitting Mg^2+^ and Co^2+^ from the reaction buffer weakened this *trans*-ssDNA cleavage activity, whereas adding a divalent ion chelating agent (EDTA) abolished the activity (Figure S6). Therefore, we named this protocol Cas3-Operated Nucleic Acid detectioN (CONAN) (Figure 1A).

### CONAN: *in vitro* nucleic acid-detection platform

We then investigated the detection sensitivity of CONAN by diluting the hEMX1 or *mTyr* dsDNA activator with Cas3, Cascade, and FQ-ssDNA in the reaction buffer. CONAN’|'s limit of detection (LoD) was >1.0 × 10^10^ copies for the activator (Figure S7). To improve the LoD, we performed isothermal RPA (TwistAmp Basic kit, Maidenhead, UK) at 37 °C for 30 min, followed by a 10-min incubation with Cascade and Cas3, thereby determining the activator’|'s single-copy sensitivity level (∼1.7 a.m.) (Figure 1C). We also achieved robust detection of the attomolar (aM) level activator by CONAN after mixing it with mouse genomic DNA (Figure 1D). Detecting FQ-ssDNA cleavage needs a microplate reader for fluorescence intensity measurement. Instead of this laboratory instrument, a lateral flow strip can be used for instrument-free and portable diagnosis by the virus POCT (Kellner et al., 2019; Lucia et al., 2020). In principle, abundant reporter accumulates anti-FITC antibody-gold nanoparticle conjugates at the first line (negative) on the strip, whereas cleavage of the reporter would reduce accumulation on the first line and result in signal on the second line (positive) with <2 min of flow (Figure S8). Using this lateral flow strip we performed a one-pot assay with CONAN-RPA, thereby detecting a single copy level of the target dsDNA within 1 hour (Figure 1D).

### CONAN-based assay for rapid detection of SARS-CoV-2 and influenza virus

**Figure 2:**
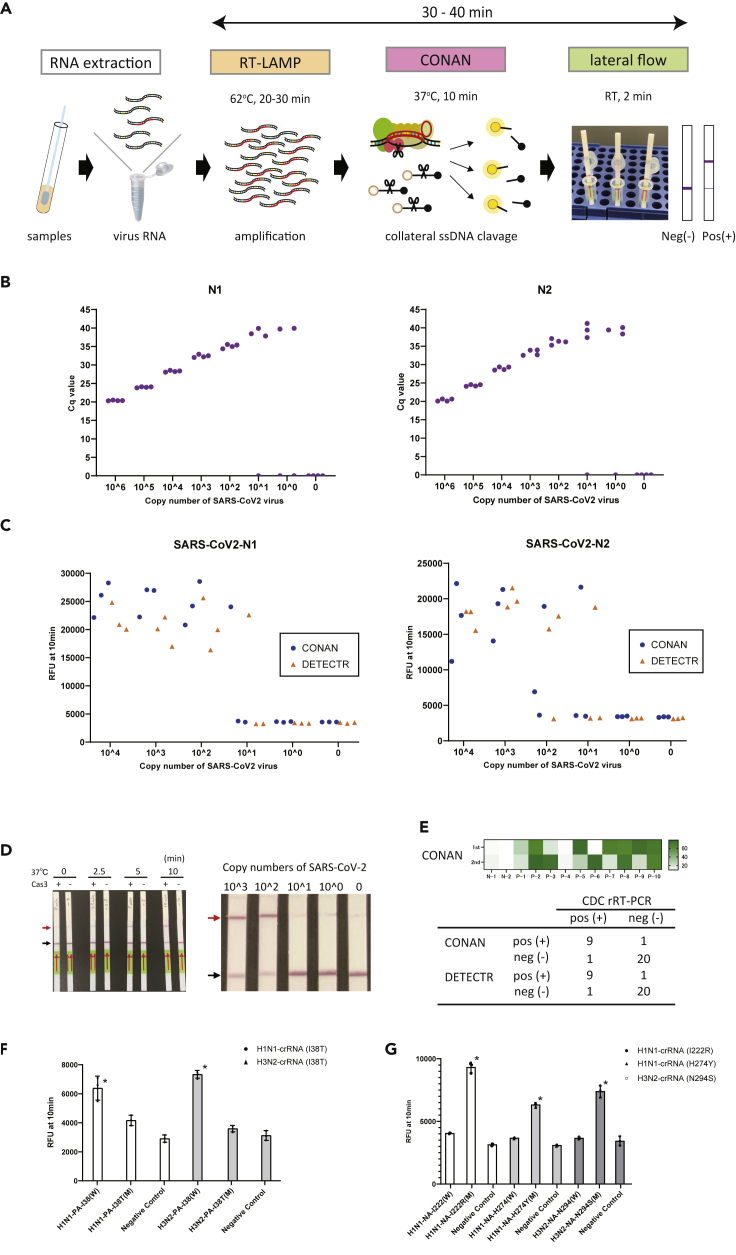
CRISPR-Cas3-based assay for rapid detection of SARS-CoV-2 and influenza virus (A) Schematic representation of the CONAN SARS-CoV-2 detection assay including a conventional RNA extraction step, RT-LAMP (62 °C, 20–30 min), CONAN (37 °C, 10 min), and lateral flow (RT, 2 min). (B) The limit of detection (LoD) of the US CDC’|'s RT-qPCR assay amplification of the N1 and N2 regions of the SARS-CoV-2 N gene. Cq, cycle quantification value. (C) LoD of CONAN-based and DETECTR-based assays for the N1 and N2 region of SARS-CoV-2. RFU, relative fluorescence unit. (D) LoD of the CONAN-based lateral flow assay for the N1 region of SARS-CoV-2. CONAN’|'s LoD was 2 min incubation (left) and <10^2^ copies (right). Positive (red arrow) and negative (black arrow) bands for CONAN (see Figure S8). (E) Comparison of SARS-CoV-2 CONAN and DETECTR assays on 31 clinical samples (10 positive and 21 negative for SARS-CoV-2 by the CDC RT-qPCR assay) (see also Figure S10). (F) CONAN-based assay for detecting I38T variants in influenza viruses. Means (n = 3) and standard deviations. ∗p < 0.01, one-way ANOVA with *post-hoc* test. (G) CONAN-based assay for detecting I222R, H274Y, and N294S variants in influenza viruses. Means (n = 3) and standard deviations. ∗p < 0.01, one-way ANOVA with *post-hoc* test.

We next examined whether the CONAN lateral flow assay would be effective for SARS-CoV-2 diagnosis (Figure 2A), as has been recently reported for DETECTR (Broughton et al., 2020; Ding et al., 2020; Lucia et al., 2020) and for SHERLOCK (Ackerman et al., 2020; Metsky et al., 2020). We designed primers to amplify the N (nucleoprotein) gene regions (N1 and N2) from SARS-CoV-2 (Table S3), which overlap with the region used in the DETECTR-based assay (Broughton et al., 2020), along with primers for the RT-qPCR assay from the United States Centers for Disease Control (US CDC) (Kimball et al., 2020). The RT-qPCR assay successfully amplified both the N1 and N2 regions of SARS-CoV-2, with LoDs of <10^2^ copies (Figure 2B). However, RT-RPA followed by Cas3-based CONAN or Cas12a-based DETECTR in the one-step 37 °C 30 min incubation (Table S1) did not effectively detect SARS-CoV-2, probably because the N1 and N2 primers we designed for RT-RPA (Table S3) did not match the sensitivity of the CRISPR-based assay (Figure S9).

Designing primers for LAMP assays can be complicated, but the upside is that these assays seem to be less sensitive to inhibitors or off-target nucleic acid contamination in the samples (Francois et al., 2011; Notomi et al., 2000). RT and LAMP at 62 °C for 30 min, followed by CONAN or DETECTR for 10 min at 37 °C, both specifically detected SARS-CoV-2 with crRNA–N1 and –N2 (Figure 2C). The LoDs for CONAN-LAMP and DETECTR-LAMP (<10^2^ copies) compared favorably with the CDC’|'s RT-qPCR assay for SARS-CoV-2 detection (Broughton et al., 2020; Corman et al., 2020; Udugama et al., 2020). This rapid detection by CONAN-LAMP was achieved at 62 °C for 30 min, with the lateral flow strip achieving a LoD of <10^2^ copies for SARS-CoV-2 (Figure 2D and Video S1).

Video S1. CONAN-based assay with lateral flow strip readouts for rapid detection of SARS-CoV-2, related to Figure 2D

We then tested the extracted RNA samples from 10 PCR-positive COVID-19 patients and 21 PCR-negative samples from nasopharyngeal swabs, using the CONAN RT-LAMP and DETCTR RT-LAMP assays with lateral flow strip readouts (Figures 2E and S10). SARS-CoV-2 was detected by the CONAN RT-LAMP assay in nine of 10 patient swabs and detected in one of 21 negative swab samples (positive predictive agreement, 90%; negative predictive agreement, 95%). One negative swab from a COVID-19 patient was confirmed to be below the established LoD of <10^2^ copies (Figure S10). This 94% detection rate (29/31) is comparable with that of the DETECTR RT-LAMP assay in this study (Figure 2E) and that previously reported for a COVID-19 POCT (Broughton et al., 2020).

Finally, we investigated whether CONAN was able to discriminate a single-base-pair mutation in the target sequences, as previously shown by Cas13 SHERLOCK (Gootenberg et al., 2017, 2018) and by Cas12b CDetection (Teng et al., 2019). The I38T drug-resistance mutation in influenza virus A confers reduced susceptibility to baloxavir treatment for IAV infections (Imai et al., 2020; Sato et al., 2020). We designed a set of crRNAs for CONAN specifically targeting the I38T variant in IAV H1N1pdm09 and H3N2 strains (Table S1). After incubation, the CONAN assay clearly only detected wild-type (WT) H1N1pdm09 and H3N2 strains, but not I38T variants (Figure 2F). To further evaluate the specificity of single-base-pair discrimination by CONAN, several crRNAs were also designed to detect the neuraminidase mutations that reduce viral susceptibility to oseltamivir in the IAV H1N1pdm09 strain (I222R and H274Y) and in the IAV H3N2 strain (N294S) (Hatakeyama et al., 2007; McKimm-Breschkin, 2013) (Table S1). Again, the CONAN assay specifically identified all three mutations (encoding I222R, H274Y, and N294S), but not the corresponding WT positions (Figure 2G). Thus, the accurate SNP-level detection by CONAN should facilitate quick and mobile POCT in hospitals and other medical facilities.

## Discussion

In this study, we reported on the discovery of Class I Cas3-mediated collateral *trans* cleavage activity. Our newly developed CONAN lateral flow assay, which uses this type of collateral cleavage, facilitated the rapid, robust, and sensitive detection of the novel coronavirus, SARS-CoV-2. Unlike the Class II single Cas12 and Cas13 platforms, CONAN employs multiple Cas proteins (Cas3, 5, 6, 7, 8, and 11), which can be premixed in the reaction buffer. By screening mismatches for each nucleotide in the 32-nt spacer, we found that a single mismatch in the spacer region, even within the seed region (positions 1–8), resulted in little or no effect on collateral ssDNA cleavage activity (Figure S11 and Table S4). This is supported by recently reported results showing that Cas12a has multiple nicking activities with tolerance of four to eight mismatches within the spacer sequences (Fu et al., 2019; Murugan et al., 2020). In contrast, CONAN shows high specificity for single-base-pair discrimination within the PAM site, supporting the applicability of CONAN-based detection assays for POCTs, even on novel emerging coronavirus mutants such as the A,B,C types (Forster et al., 2020) as well as spike protein mutations (Korber et al., 2020).

In summary, we have shown that CONAN, a Cas3-based novel *in vitro* nucleic acid-detection platform, is a rapid (within 30–40 min), low-cost, and instrument-free detection method for SARS-CoV-2. We also showed that this CONAN-based assay enables single-base-pair discrimination (Figures 2F and 2G) (Yoshimi et al., 2021), thereby facilitating the deployment of CRISPR-dx for quick and mobile POCTs for drug-resistant IAV variants in hospitals or clinics.

### Limitations of the study

Although we have discovered the collateral ssDNA cleavage activity in the EcoCascade-Cas3 system, this study does not describe the mechanisms underlying how Cas3 mediates the collateral ssDNA cleavages as well as targeted double-stranded DNA cleavages. Our preprint manuscript describes several insights for these molecular mechanisms (Yoshimi et al., 2021).

## STAR★Methods

### Key resources table

**Table:** 

REAGENT or RESOURCE	SOURCE	IDENTIFIER
**Bacterial and virus strains**		

*E. coli*: MAX Efficiency™ DH5α Competent Cells	Thermo Fisher Scientific	Cat#18258012
*E. coli*: MAX Efficiency™ DH10Bac Competent Cells	Thermo Fisher Scientific	Cat#10361012
*E. coli*: JM109(DE3)	Promega	Cat#P980A

**Biological samples**		

SARS-CoV-2 clinical samples	IMSUT hospital	N/A
*E.coli* Cascade-crRNA complex, see Table S1	In-house	N/A
*E.coli* Cas3 protein	In-house	N/A
LbCas12a (Cpf1) protein	Integrated DNA Technologies	Cat#10007922

**Chemicals, peptides, and recombinant proteins**		

ATP	TaKaRa	Cat#4041
BpiI (BbsI)	Thermo Fisher Scientific	Cat#ER1011
DNA ligation mix	TaKaRa	Cat#6023
Tks Gflex™ DNA polymerase	TaKaRa	Cat#R060A
ExoSAP-IT™ Express PCR product cleanup reagent	Applied Biosystems	Cat#75001.1.ML
HEPES-KOH pH 7.5	NACALAI TESQUE	Cat#15639-84
Ni-NTA agarose	Qiagen	Cat#30210
Superdex 200 Increase 10/300 GL	Cytiva	Cat#28990944
PSFM-J1 medium	Fujifilm-Wako	Cat#160-25851
FBS	Thermo Fisher Scientific	Cat#10270-106
2-YT Broth	Thermo Fisher Scientific	Cat #22712020
IPTG (Isopropyl-β-D-thiogalactopyranoside)	TaKaRa	Cat#9030
FuGENE® HD transfection reagent	Promega	Cat#E2311

**Critical commercial assays**		

PureLink HiPure plasmid filter midiprep kit	Thermo Fisher Scientific	Cat#K210015
TwistAmp Basic	TwistDX	Cat#TABAS03KIT
WarmStart LAMP kit (DNA & RNA)	NEB	Cat#E1700S
HybriDetect – universal lateral flow assay kit	Milenia Biotec	Cat#MGHD 1
SARS-CoV-2 direct detection RT-qPCR kit	TaKaRa	Cat#RC300A
Bac-to-Bac™ vector kit	Thermo Fisher Scientific	Cat#10360014

**Experimental models: cell lines**		

VeroE6/TMPRSS2	Japanese Collection of Research Bioresources Cell Bank	https://cellbank.nibiohn.go.jp/english/ no. JCRB1819
Sf9 cells	Thermo Fisher Scientific	Cat#11496015

**Oligonucleotides**		

Primers, see Table S3	Eurofins Genomics	N/A
Primers with quenchers and probes, see Table S3	Integrated DNA Technologies	N/A

**Recombinant DNA**		

pRSFDuet-1	Merck Millipore	Cat#71341-3CN
pCDFDuet-1	Merck Millipore	Cat#71340-3CN
pACYCDuet-1	Merck Millipore	Cat#71147-3CN
pACYCDuet-1_crRNA-empty-Bbs1v2	In-house	N/A
pCDFDuet-1_6His-Cas11	In-house	N/A
pRSFDuet-1_ Cas5-6-7-8-11	In-house	N/A
pFastbac-1_8HisCas3	In-house	N/A

**Software and algorithms**		

ImageJ	(Schneider et al., 2012)	https://imagej.nih.gov/ij/
Prism 8	GraphPad	https://www.graphpad.com/scientific-software/prism
PrimerExplorer v.5	Eiken Chemical	https://primerexplorer.jp/

**Other**		

Thermal cycler	Bio-rad	Cat#T100
qPCR instruments	Bio-rad	Cat#CFX Connect
New Brunswick™ Innova® 44R bioshaker	Eppendorf	Cat#M1282-0007
BR-43FL MR bioshaker	TAITEC	Cat#0053027-000
Cool Incubator i-CUBE	AS ONE	Cat#FCI-280HG
AKTA pure 25	Cytiva	Cat#29018225

### Resource availability

#### Lead contact

Further information and requests for resources and reagents should be directed to and will be fulfilled by the Lead Contact, Tomoji Mashimo (mashimo@ims.u-tokyo.sc.jp).

#### Materials availability

All reagents and materials used in this manuscript are available upon request or prepared to be available from commercial sources.

### Experimental model and subject details

#### TMPRSS2-expressing VeroE6 cells and Sf9 cells

Vero E6 cells expressing human serine protease TMPRSS2 (VeroE6-TMPRSS2), derived from the kidney of an African green monkey, were kindly provided from National Institute of Infectious Diseases. The cells are available from the Japanese Collection of Research Bioresources Cell Bank in Japan (https://cellbank.nibiohn.go.jp/english/) (JCRB no. JCRB1819). The VeroE6-TMPRSS2 cells were incubated at 37°C under 5% CO_2_ in Dulbecco's Modified Eagle Medium (DMEM) containing 10% fetal calf serum (FCS), 1 mg/ml G418, 100 units/ml penicillin, 100 μg/ml streptomycin, and 5 μg/ml plasmocin prophylactic (InvivoGen, CA, USA). Sf9 cells were obtained from Thermo Fisher Scientific (Waltham, Massachusetts, USA) and maintained at 28°C with 130 rpm in PSFM-J1 medium (Fujifilm-wako, Osaka, Japan).

#### Baculovirus

Baculoviruses for EcoCas3 protein were produced using the Bac-to-Bac™ vector kit (Thermo Fisher Scientific) following to the manufacturer’s protocol. Briefly, EcoCas3 cDNA were cloned into the pFastbac-1 plasmids, which were transformed to the MAX Efficiency™ DH10Bac Competent cells (Thermo Fisher Scientific) in LB medium. After the collection of the recombinant bacmid DNA by single colony screening, the bacmid DNA were transfected to the Sf9 cells by FuGENE HD transfection reagent in PSFM-J1 medium. We incubated the cells at 27 °C for 72 hours and collected the supernatant as a first virus stock after the centrifuge of 3,500 rpm for 5 minutes. To increase the virus titer, 15 ml of the virus stock were added to the Sf9 cells at 0.6 × 10^6^ cells/ml in 100 ml of PSFM-J1 medium. We again collected the supernatant as a baculovirus stock and used it for further experiments.

#### Collection of human clinical samples

Clinical nasopharyngeal and oropharyngeal swab samples from patients infected with SARS-CoV-2 were collected by IMSUT hospital (The University of Tokyo). Negative nasopharyngeal swabs were collected from healthy donors at IMSUT. RNA from the samples of patients and healthy donors was extracted as described in the NIID-approved protocol (input 140 μl; elution, 60 μl) using the Viral RNA mini kit (QIAGEN). These clinical specimens were discriminated by RT-PCR for positive or negative for SARS-CoV-2 infection prior to CRISPR-based assays. We used small sample size (10 positive and 21 negative) to validate the CONAN-based assays in this study, but more trials with a larger sample size will be needed before considering for clinical application.

The information about the patients and healthy individuals such as the age and gender are not available due to ethical and privacy restriction. However, we don’t anticipate the sex or age of the participants to have any influence on the results. Human samples were collected by following protocols approved by the Research Ethics Review Committee of the Institute of Medical Science, the University of Tokyo (approval number 2019–71–0201).

### Methods details

#### CRISPR preparation

Cascade/Cas3 and Cas12a target sites were based on the human EMX1 gene, the mouse Tyr gene, and the N gene from SARS-CoV-2 (Table S1). A baculovirus expression system was used to purify the EcoCas3 protein, as previously shown (Yoshimi et al., 2021). Briefly, EcoCas3 cDNA was cloned using an octa-histidine tag and a six asparagine-histidine repeat tag into pFastbac-1 plasmids. Sf9 cells were infected with baculovirus at a multiplicity of infection (MOI) of two with 2% FBS in PSFM-J1 medium at 28°C with 130 rpm for 24 h. We changed the culture temperature to 20°C with 110 rpm for 4 d. The expressed EcoCas3 protein was purified using nickel affinity resin (Ni-NTA, QIAGEN, Venlo, the Netherlands). To remove the tags, purified EcoCas3 was digested with TEV protease, and further purified by size-exclusion chromatography (Superdex 200 Increase 10/300 GL; Thermo Fisher Scientific) in 0.2 M NaCl, 10% glycerol, 1 mM DTT, and 20 mM HEPES-Na (pH 7.0). Purified EcoCas3 protein was evaluated by sodium dodecyl sulfate-polyacrylamide gel electrophoresis (SDS-PAGE) (Figure S1A).

The EcoCascade and crRNA complex were purified in accordance with previously reported methods (Hochstrasser et al., 2014; Jore et al., 2011; Yoshimi et al., 2021). Briefly, EcoCascade/crRNA ribonucleoproteins (RNPs) were expressed in JM109 (DE3) by co-transformation with three plasmids: one plasmid encoding a hexahistidine tag and HRV3C protease recognition site in the N-terminus of Cas11 (plasmid pCDFDuet-1); one plasmid containing the EcoCascade operon and the genes encoding Cas5, Cas6, Cas7, Cas8, and Cas11 (plasmid pRSFDuet-1); and the final plasmid encoding crRNA (pACYCDuet-1) (Yoshimi et al., 2021). The transformed bacteria were cultured in 2xYT medium at 37 °C with 130 rpm. After the OD600 became 0.6 to 0.8, we added IPTG (final concentration 0.4 mM) and cultured at 26 °C with 110 rpm for 16 hours. The expressed Cascade-crRNA RNPs were purified by Ni-NTA resin. After removing the hexahistidine tag using HRV3C protease, the EcoCascade/crRNA RNPs were further purified by size-exclusion chromatography in 350 mM NaCl, 1 mM DTT, and 20 mM HEPES-Na (pH 7.0) and size-evaluated by SDS-PAGE (Figure S1B). RNP sizes were found to be consistent with those of previous reports (Hochstrasser et al., 2014; Jore et al., 2011). LbCas12a were purchased from Integrated DNA Technologies (IDT, Coralville, IA). Target-specific crRNAs were also purchased from IDT.

#### DNA and RNA preparation

For the Cascade/Cas3 activator templates, DNA fragments of *hEMX1*, *mTyr* and IAV variants (which include a target site) were designed and purchased from IDT (Table S2). Total mouse genomic DNA from a C57BL/6 strain was used after purification (Maxwell RSC Cell DNA Purification Kit; Promega, Madison, Wisconsin). LAMP SARS-CoV-2 primers were designed against regions of the N gene using PrimerExplorer v.5 (Eiken Chemical Co.; https://primerexplorer.jp/). The primers used for isothermal PCR are listed in Table S3.

Viral RNAs from SARS-CoV-2 were prepared according to the established protocol from the National Institute of Infectious Diseases in Japan (Matsuyama et al., 2020). Viral RNAs were purified from an infected TMPRSS2-expressing VeroE6 cell line using the QIAamp Viral RNA Mini Kit (QIAGEN) according to the manufacturer’s protocol.

#### Real-time RT-PCR

RT-qPCR was used to determine SARS-CoV-2 RNA copy numbers using Reliance One-Step Multiplex RT-qPCR Supermix and CFX Connect (Bio-Rad Laboratories, Hercules, CA) according to the manufacturer’s protocols. N gene-specific primer and probe sets for RT-qPCR assays and the SARS-CoV-2 plasmid (positive control) were purchased from IDT (Table S3). Copy numbers of SARS-CoV-2 were determined based on the regression line (y = -3.3125x + 40.527), which was generated using the serial-diluted plasmids and the N2-primer set.

#### CONAN assay

To characterize the Cas3 collateral cleavage assays, DNA templates were added to 100 nM Cascade-crRNA complex, 250  nM Cas3 and 2.5 mM ATP in CRISPR-Cas3 system working buffer (60 mM KCl, 10 mM MgCl_2_, 10 μM CoCl_2_, 5 mM HEPES-KOH pH 7.5), as previously described (Yoshimi et al., 2021). The ssDNA reporter probe (5ʹ-/5HEX/AAGGTCGGA/ZEN/GTCAACGGATTTGGTC/3IBFQ/-3ʹ) (250 nM) was added, and the probe’s cleavage-related change in the fluorescence signal was measured every 30 s for 10 min under 37 °C incubation.

To detect DNAs, isothermal amplification and RPA were performed using the TwistAmp Basic kit (TwistDx, Maidenhead, UK) according to the manufacturer’s protocol. Template DNAs were amplified by incubation at 37 °C for 20 min. To detect RNAs, isothermal amplification by the RT-LAMP method was performed using the WarmStart LAMP kit (NEB) according to the manufacturer’s protocol. Template RNAs were reverse transcribed and amplified by incubation at 62 °C for 20 min. To detect low-copy-number molecules in the patients’ samples, the incubation time was extended to 45 min.

The CRISPR-Cas3 system reaction mixture used for the CONAN method contained 100 nM EcoCascade-crRNA complex, 400  nM Cas3 and 2.5 mM ATP in the working buffer. LbCas12a were prepared as described previously^12^ for use as positive controls for *trans* cleavage activity. Cas12a (50  nM) was incubated with 62.5  nM of crRNA in 1× NEBuffer 2.1 for 30  min at 37 °C. After amplification, 2 μl of the amplicon was combined with 18 μl of Cas3 and the Cascade-crRNA complex or the Cas12a-crRNA complex, and 250 nM of the ssDNA reporter probe was added. The fluorescence signal was measured every 30 s at 37 °C.

#### Lateral flow assay

To optimize the CONAN assay for lateral flow readouts, the CRISPR-Cas3 system reaction mixture was added to 200 nM of the Cascade-crRNA complex, with 400 nM Cas3 and 2.5 mM ATP in the working buffer. A 2 μl aliquot of the amplicon was added to 18 μl Cas3, Cascade-crRNA complex and 500 nM ssDNA reporter probe (5ʹ-/5-FITC/TAGCATGTCA/3-Biotin/-3ʹ). The mixture was incubated for 10 min at 37 °C. After adding 50 μl nuclease-free water, a lateral flow strip (Milenia HybriDetect 1; TwistDx) was added to the reaction tube and the result was visualized after approximately 2 min. A lower band close to the sample pad indicated a negative result (uncut probes), whereas an upper band close to the top of the strip indicated the 5′ end of the cut probes. Emergence of an upper band indicated a positive result (Figure S8). The test band intensities on the lateral flow strips were quantified by the gray values using the ImageJ tool and were visualized on a heat map (Figure 1E).

### Quantification and statistical analysis

All statistical analyses were performed by using GraphPad Prism 8 software. Graph data are presented as mean ± SD. Statistical details of the analyses can be found in the figure legends.

## Data Availability

This study did not generate any unique datasets or code. Any additional information required to reanalyze the data reported in this work paper is available from the Lead Contact upon request.
