# Supplementary material for: CRISPR-Cas3-based diagnostics for SARS-CoV-2 and influenza virus
Source: iScience. 2022 Jan 30;25(2):103830. doi: 10.1016/j.isci.2022.103830 (PMC8801231; doi:10.1016/j.isci.2022.103830)
Supplement: Document S1. Figures S1–S11 and Tables S1–S4 [file mmc1.pdf]

iScience, Volume *25*

Supplemental information

CRISPR-Cas3-based diagnostics

for SARS-CoV-2 and influenza virus

Kazuto Yoshimi, Kohei Takeshita, Seiya Yamayoshi, Satomi Shibumura, Yuko Yamauchi, Masaki Yamamoto, Hiroshi Yotsuyanagi, Yoshihiro Kawaoka, and Tomoji Mashimo

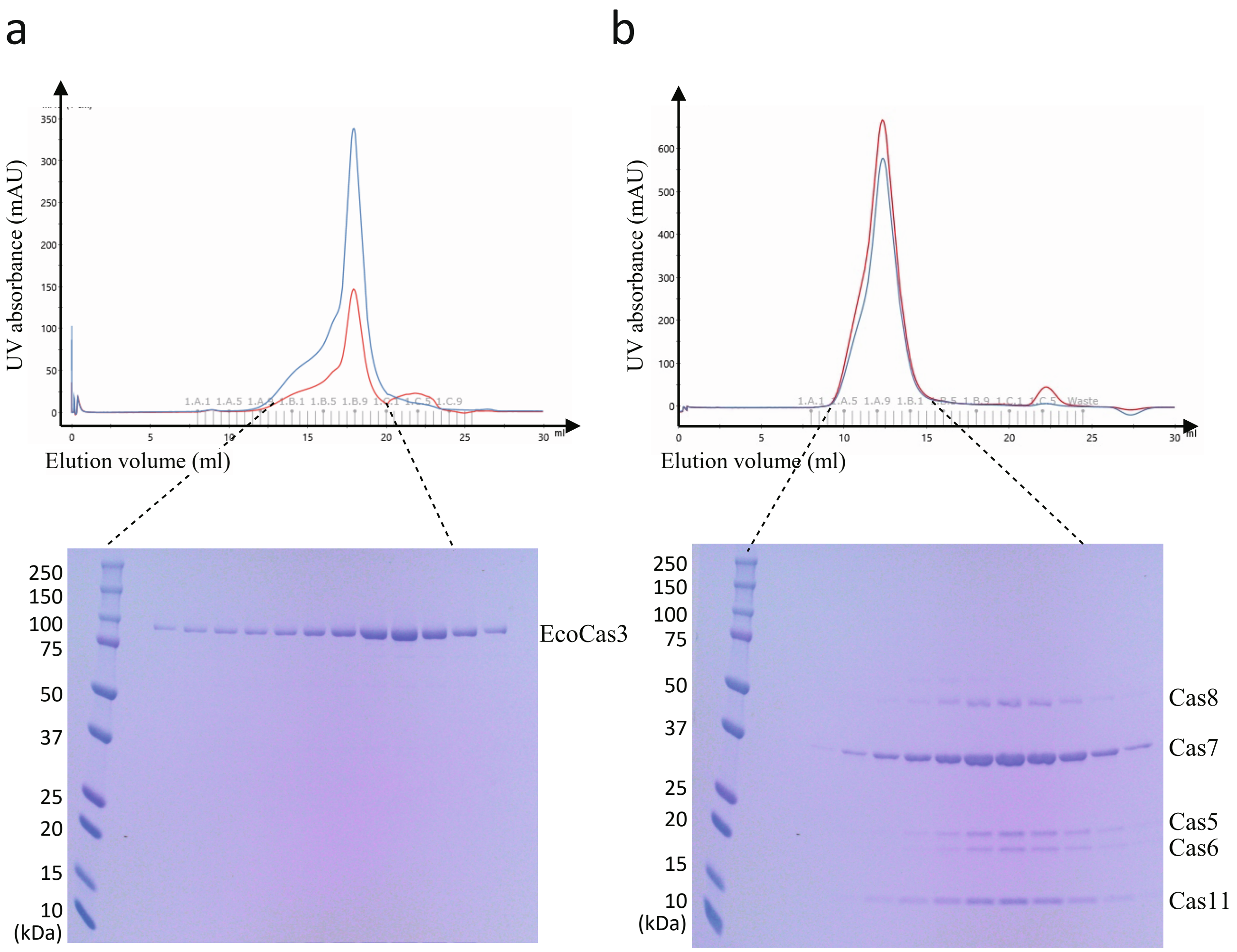

Fig. S1. Size-exclusion chromatography and SDS-PAGE for EcoCas3 and EcoCascade (Cas5, Cas6, Cas7, Cas8 and Cas11) ribonucleoproteins. Related to STAR Methods. (a) A massive amount of EcoCas3 protein was expressed using the baculovirus expression system in Sf9 insect cells cultured at 20 degree. Purified EcoCas3 protein was soluble, and ~95 % homogeneous in SDS-PAGE. (b) A complex of Cas5, Cas6, Cas7, Cas8 and Cas11 proteins and crRNA was co-expressed in JM109(DE3) *E.coli* cultured at 26 degree.

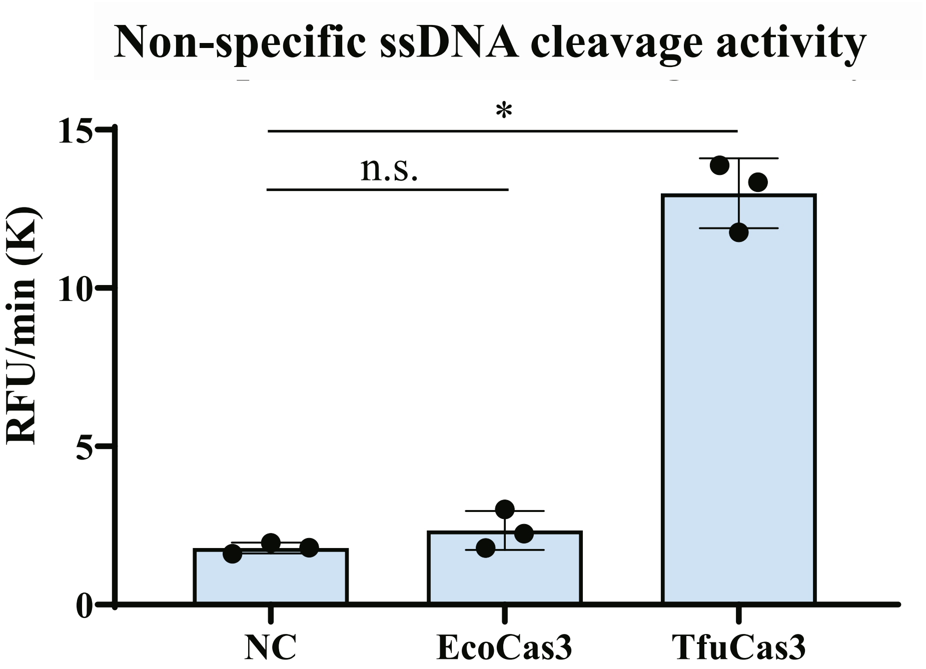

Fig. S2. Non-specific ssDNA cleavage activity by Cas3 proteins. Related to STAR Methods. Incubating Cas3 derived from *T. fusca* (TfuCas3) with FQ-ssDNA probes resulted in non-specific ssDNA cleavage activity, but no activity by that derived from *E. coli* (EcoCas3). RFU/min: increasing rate of relative fluorescence units/min. NC: negative control. Means (n=3) and standard deviations. *:p<0.001 n.s.: not significant.

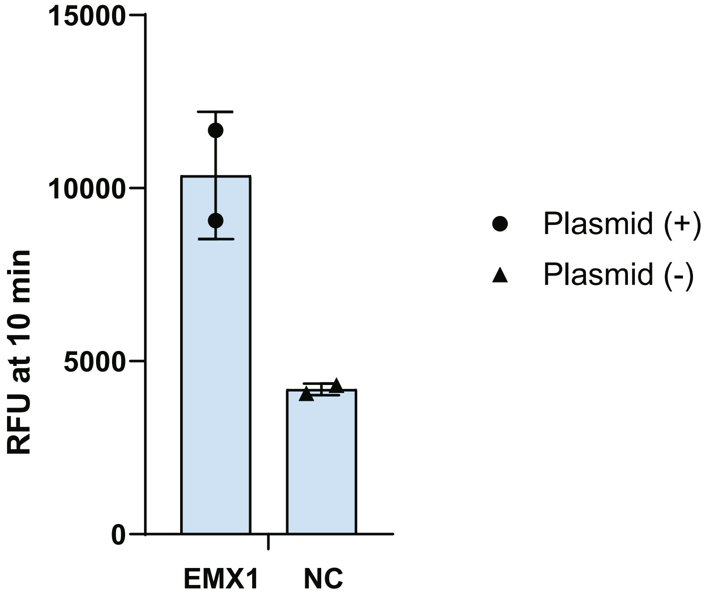

Fig. S3. Collateral cleavage activity by CONAN. Related to Fig. 1b. The 3-Kb dsDNA plasmid (+), which includes the target *hEMX1* sequence and the EcoCas3/EcoCascade-crRNA, activates *trans* cleavage of the FQ-ssDNA probe. NC: negative control (plasmid (-)). RFU: relative fluorescence units at 10 min post-incubation. Means (n=2) and standard deviations.

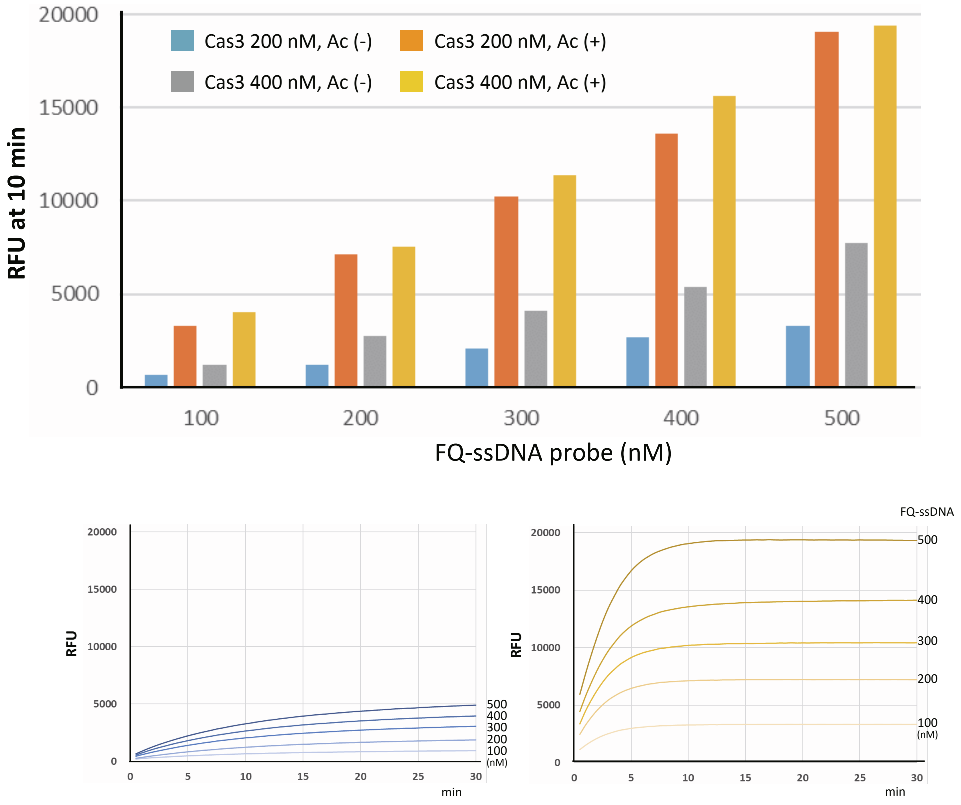

Fig. S4. Collateral cleavage activity by CONAN. Related to Fig. 1b.
The concentration of the FQ-ssDNA probe (nM) correlated with the signal intensity (RFU), suggesting that Cas3 cleaves several copies of ssDNA per bound Cascade. EcoCas3 (200 or 400 nM), EcoCascade-crRNA complex (200 nM), 60-bp ddsDNA activator for EMX1 (Ac), and FQ-labeled ssDNA probe were in the reaction buffer. RFU: relative fluorescence unit.

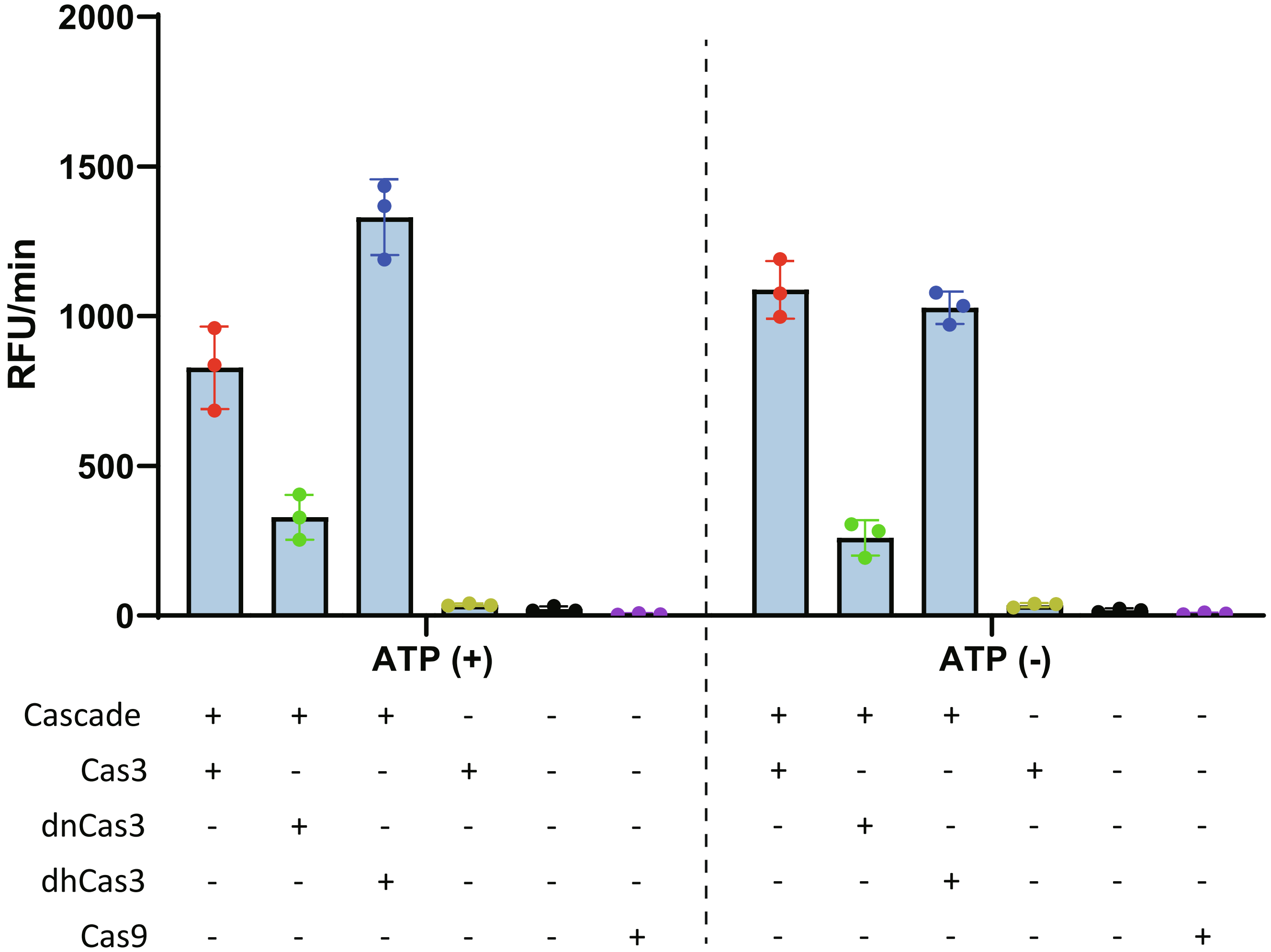

Fig. S5. Collateral cleavage activity by CONAN. Related to Fig. 1b. EcoCas3 HD domain H74A (dead nuclease mutant, dnCas3), abolished collateral cleavage activity, while SF2 motif III S483A/T485A (dead helicase mutant, dhCas3) showed collateral cleavage activity. Collateral activity in ATP reaction buffer (+) was at the same level as that in ATP-free buffer (-) for wild-type EcoCas3 and the dhCas3 mutant. SpCas9 did not exhibit any collateral cleavage activity.

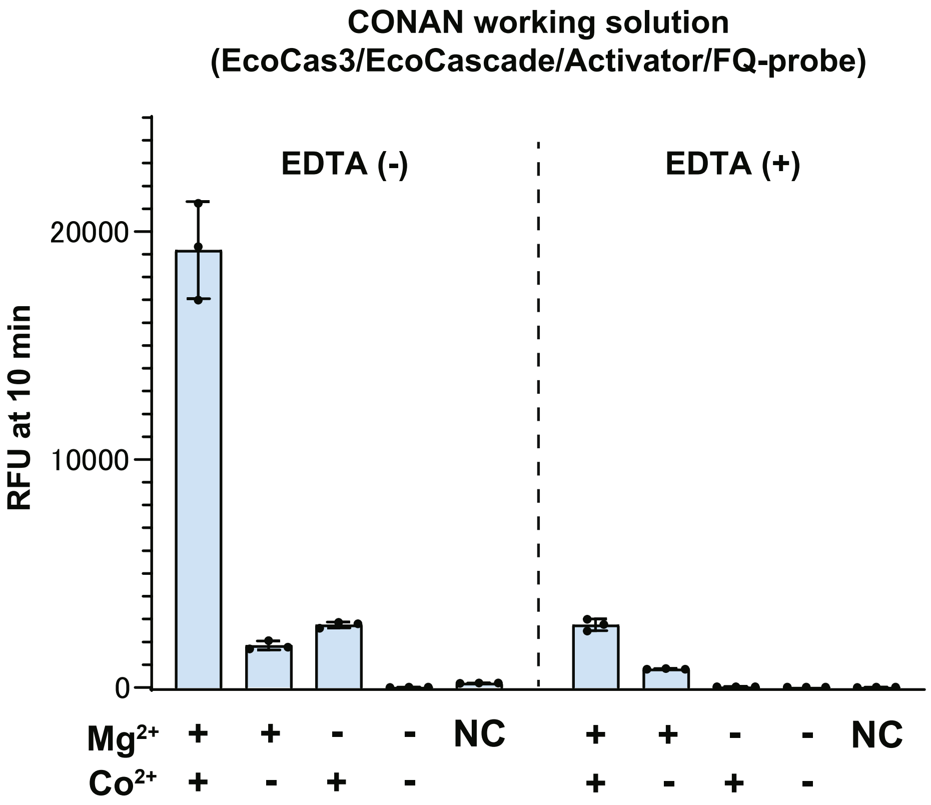

Fig. S6. Collateral cleavage activity by CONAN. Related to Fig. 1b. Absence of Mg^{2+} and Co^{2+} in the reaction buffer weakened the *trans*-ssDNA cleavage activity. Presence of a divalent ion chelating agent, EDTA (+), abolished the collateral cleavage activity. RFU: relative fluorescence unit. Means (n=3) and standard deviations.

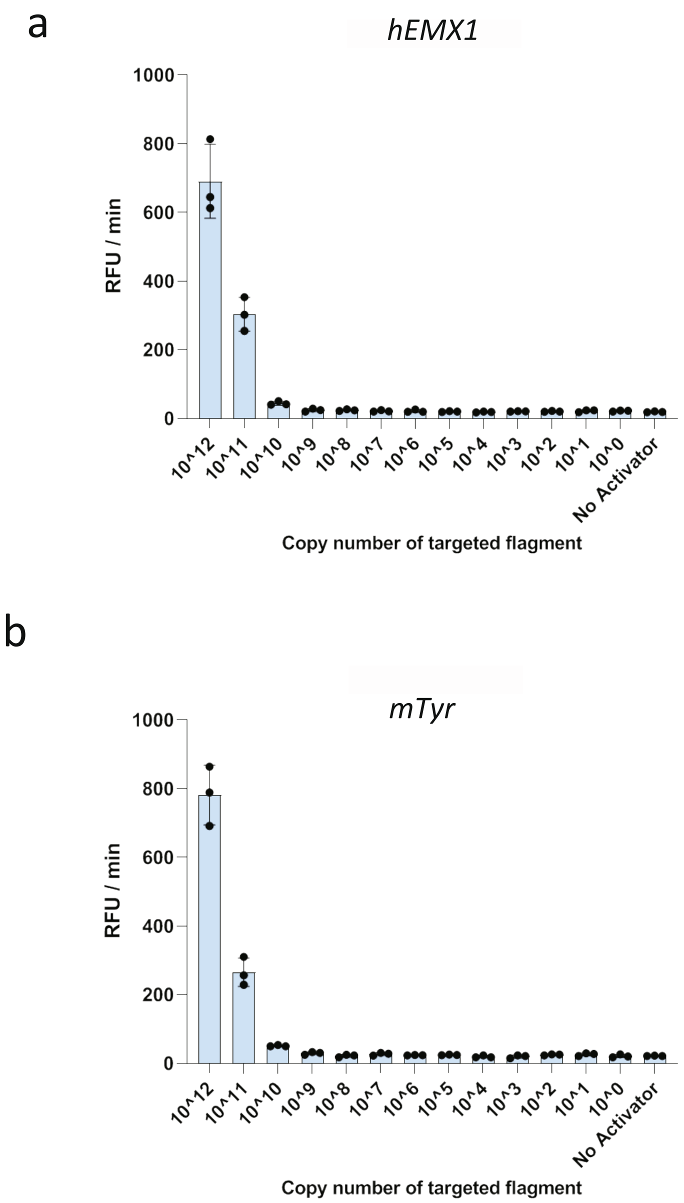

Fig. S7. Limit of detection (LoD) of the CONAN assay. Related to Fig. 1c. Dilution of the dsDNA activator *hEMX1* (a) or mouse genome dsDNA *mTyr* (b) with Cas3, Cascade, and FQ-ssDNA in the reaction buffer. RFU/min: increasing rate of relative fluorescence unit/min. Means (n=3) and standard deviations.

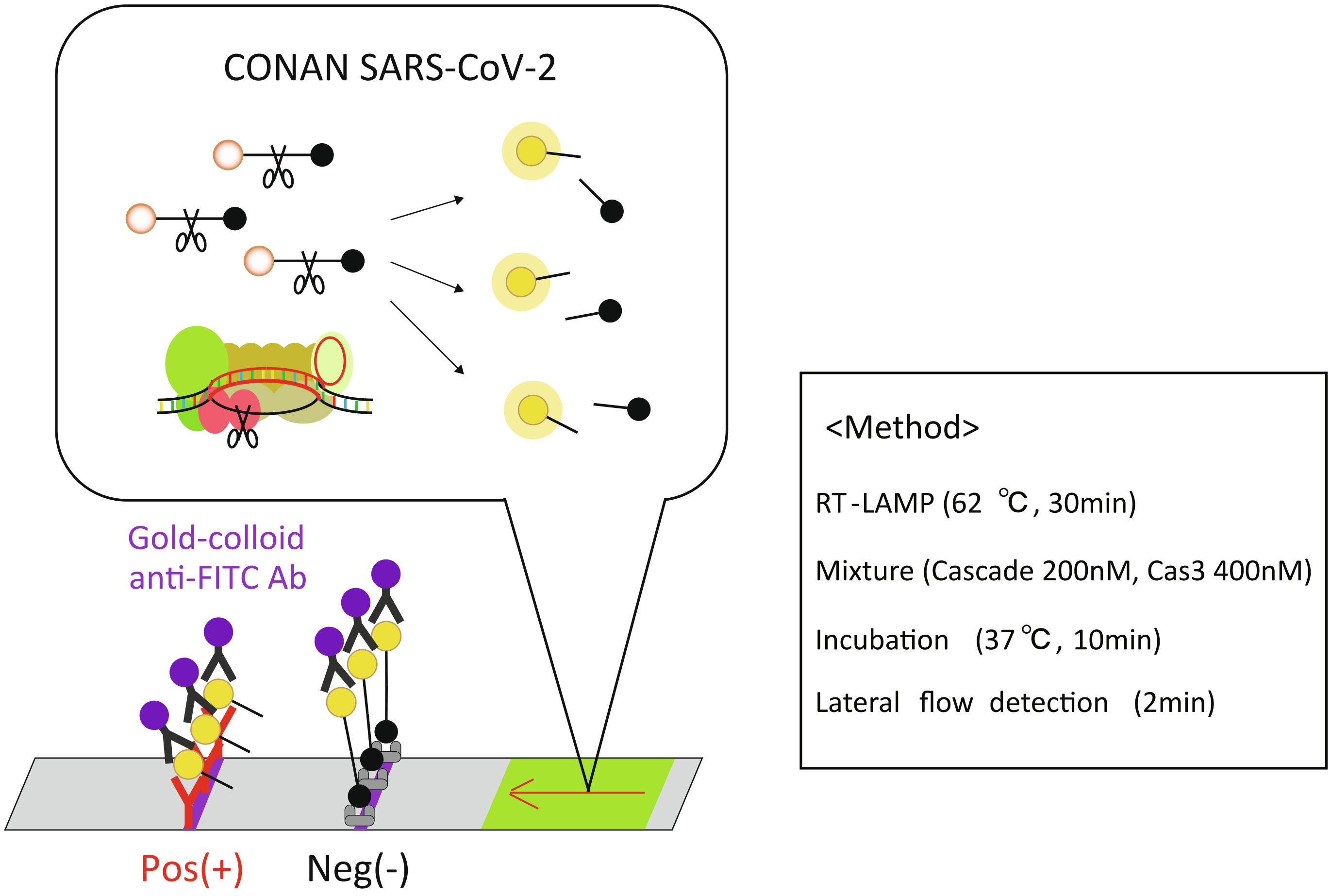

Fig. S8. Lateral-flow detection with CONAN. Related to Fig. 2d and STAR Methods. Abundant uncleaved FQ-labeled ssDNA reporter accumulates anti-FAM antibody-gold nanoparticle conjugates at the first line (negative) on the strip, while cleaved reporter reduce accumulation at the first line and result in signal on the second line (positive) for <2 min flow.

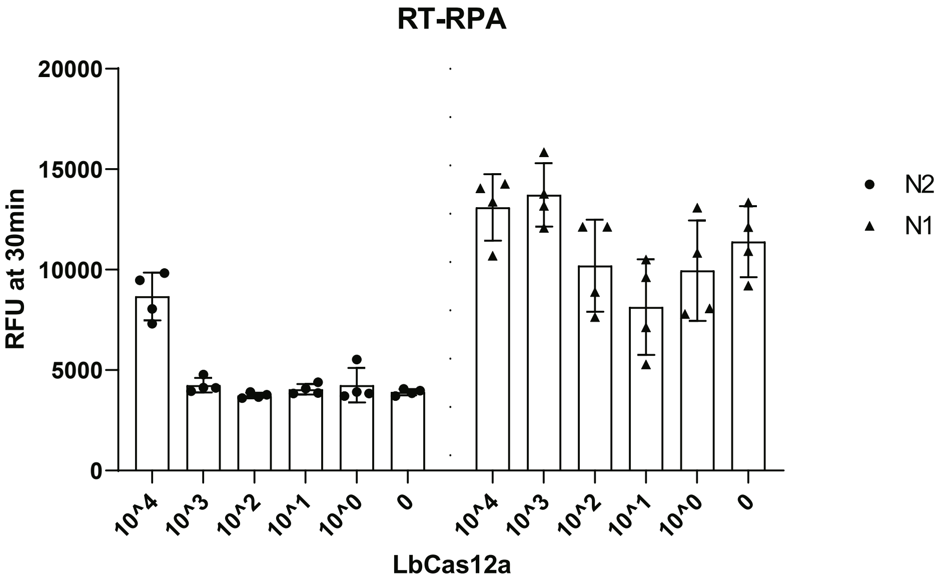

Fig. S9. Limit of detection (LoD) of the Cas12a-based DETECTR RT-RPA assay using N1 and N2 primers designed for the N gene region of SARS-CoV-2. Related to Fig. 2b and Table S1. RT-RPA followed by Cas12a-based DETECTR in the one-step 37ºC 30-min incubation did not effectively detect SARS-CoV-2. RFU: relative fluorescence at 30 min. Means (n=3) and standard deviations.

Sample name	Ct value (5 µL sample)	Estimated Copy number (/µL)	Image of a lateral flow assay 1st	Image of a lateral flow assay 2nd	CONAN detection (Glay depth) 1st		CONAN detection (Glay depth) 2nd		Result
NP- (N1)	-	-			11.1	-	12.8	-	Negative Control
NP- (N2)	-	-			10.2	-	7.0	-	Negative Control
NPCo-015NT1 (P1)	24.51	13688			27.0	+	19.7	-	Positive (Intermediate)
NPCo-016NT1 (P2)	23.31	31522			63.3	+	78.7	+	Positive
NPCo-018NT1 (P3)	28.56	820			18.7	-	73.5	+	Positive (Intermediate)
NPCo-021NT1 (P4)	34.64	12			11.0	-	12.3	-	Negative
NPCo-021NT2 (P5)	33.64	24			75.7	+	27.0	+	Positive
NPCo-022NT1 (P6)	30.49	214			8.3	-	77.2	+	Positive (Intermediate)
NPCo-023NT1 (P7)	34.73	11			65.3	+	19.3	-	Positive (Intermediate)
NPCo-025NT1 (P8)	28.60	797			62.7	+	71.2	+	Positive
NPCo-025NT2 (P9)	26.91	2581			74.4	+	22.0	+	Positive
NPCo-026NT1 (P10)	25.37	7529			69.0	+	64.8	+	Positive

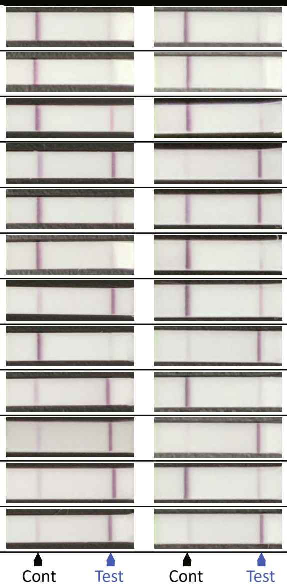

Fig. S10. SARS-CoV-2 CONAN lateral flow assay results for 10 PCR-positive samples. Related to Fig. 2e. We tested the extracted RNA samples from 10 PCR-positive patients with COVID-19 (P1–10) and PCR-negative samples (N1-2) from nasopharyngeal swabs using CONAN RT-LAMP with lateral flow strip readouts. In CONAN detection, the gray depth of the second test line (blue) is measured after 2 min of flow.

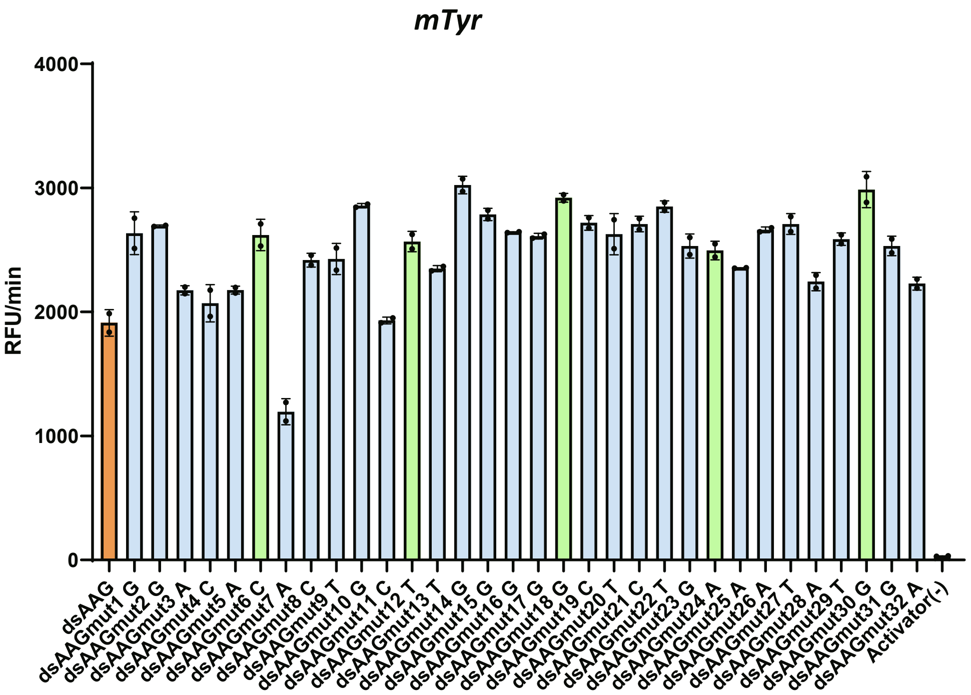

Fig. S11. The effect of mismatch of each 32-nt spacer sequence on collateral cleavage activity. Related to Fig. 2f, g and Table S4. A single mismatch in the spacer region has a little or no effect on collateral cleavage activity of *mTyr* target.

Table S1. Target sequences used for the trans cleavage assay. Related to Fig. 1, 2, S9, and STAR Methods.

Name	PAM (5' to 3')	Sequence (5' to 3')
CRISPR-Cas3		
hEMX1	AAG	CAGGCCAATGGGGAGGACATCGATGTCACCTC
mTyr	AAG	GGACACACTGCTTGGGGGCTCTGAAATATGGA
SARS-N1	AAG	GCCAAACTGTCACTAAGAAATCTGCTGCTGAG
SARS-N2	AAG	GAACTGATTACAAACATTGGCCGCAAATTGCA
IAV-H1N1-I38	AAT	TGCAGCAAACTTATTAGTTTCAATTTTGGGGT
IAV-H3N2-I38	ATA	TGCACTCACTTGGAGGTGTGTTTCATGTATTC
IAV-H1N1-I222-Mu	TAG	ATTGAGAACACAAGAGTCTGAATGTGCATGTG
IAV-H1N1-H274-Mu	TAA	TAATTAGGGGCTTTCATTTCGACTGATTTGAT
IAV-H3N2-N294-Mu	CAG	CTGTCTCTGCAGACACATCTGACACCAGGATA
CRISPR-Cas12a		
hEMX1	TTTG	TGGTTGCCCACCCTAGTCATT
SARS-N1	TTTC	TTAGTGACAGTTTGGCCTTG
SARS-N2	TTTG	CCCCCAGCGCTTCAGCGTTC

Table S2. DNA fragment sequences used for CONAN. Related to Fig. 1, 2, and STAR Methods.

Name	Sequence (5' to 3')
EMX1-AAG	TGGCGCATTGCCACGAAGCAGGCCAATGGGGAGGACATCGATGTCACCTCCAATGACTAG
mTyr-AAG	GCATTACTATGTGTCAAGGGACACACTGCTTGGGGGCTCTGAAATATGGAGGGACATTGA
H1N1pdm09-PA-I38	GGGAAGACCCCAAAATTGAAACTAATAAGTTTGCTGCAATTTGCACACATTTGGAAGTTTGTTTCATGTATTCGGATTTC
H1N1pdm09-PA-I38T	GGGAAGACCCCAAAATTGAAACTAATAAGTTTGCTGCAACTTGCACACATTTGGAAGTTTGTTTCATGTATTCGGATTTC
H1N1pdm09-NA-I222	GGCATAATAACAGACACTATCAAGAGTTGGAGGAACAATATATTGAGAACACAAGAGTCTGAATGTGCATGTGTAAATGG
H1N1pdm09-NA-I222R	GGCATAATAACAGACACTATCAAGAGTTGGAGGAACAATAGATTGAGAACACAAGAGTCTGAATGTGCATGTGTAAATGG
H1N1pdm09-NA-H274	AAAGATAATCAAATCAGTCGAAATGAAAGCCCCTAATTATCACTATGAGGAATGCTCCTGTTACCCTGATTCTAGTGAAA
H1N1pdm09-NA-H274Y	AAAGATAATCAAATCAGTCGAAATGAAAGCCCCTAATTATTACTATGAGGAATGCTCCTGTTACCCTGATTCTAGTGAAA
H3N2-PA-I38	GGGGAGGATCTGAAAATTGAAACCAACAAATTTGCAGCAATATGCACTCACTTGGAGGTGTGTTTCATGTATTCAGATTT
H3N2-PA-I38T	GGGGAGGATCTGAAAATTGAAACCAACAAATTTGCAGCAACATGCACTCACTTGGAGGTGTGTTTCATGTATTCAGATTT
H3N2-NA-N294	TATCCTCGATATCCTGGTGTCAGATGTGTCTGCAGAGACAACTGGAAAGGATCCAACCGGCCCATCATAGATATAAACAT
H3N2-NA-N294S	TATCCTCGATATCCTGGTGTCAGATGTGTCTGCAGAGACAGCTGGAAAGGATCCAACCGGCCCATCATAGATATAAACAT

Table S3. Primer sequences used for isothermal PCR. Related to Fig. 1, 2, and STAR Methods.

Name	Sequence (5' to 3')
for qRT-PCR	
SARS-N1-qPCR-F	GACCCCAAAATCAGCGAAAT
SARS-N1-qPCR-R	TCTGGTTACTGCCAGTTGAATCTG
SARS-N1-qPCR-probe	ACCCCGCATTACGTTTGGTGGACC
SARS-N2-qPCR-F	TTACAAACATTGGCCGCAAA
SARS-N2-qPCR-R	GCGCGACATTCCGAAGAA
SARS-N2-qPCR-probe	ACAATTTGCCCCCAGCGCTTCAG
for RPA	
EMX1-F	GAAGAAGGGCTCCCATCACATCAACCGGTG
EMX1-R	GCCAGCAGCAAGCAGCACTCTGCCCTCGTG
SARS-N1-F	CTTGCTTTGCTGCTGCTTGACAGATTGAAC
SARS-N1-R	TTGTTCTGGACCACGTCTGCCGAAAGCTTG
SARS-N2-F	TTCGGCAGACGTGGTCCAGAACAAACCCAA
SARS-N2-R	CCTGTGTAGGTCAACCACGTTCCCGAAGGT
for RT-LAMP	
<SARS-N1 set>	
SARS-N1-FIP	GGTTCAATCTGTCAAGCAGCAGTAGAATGGCTGGCAATGG
SARS-N1-BIP	AAGAAGCCTCGGCAAAAACGGTTTGTTCTGGACCACGT
SARS-N1-F3	AGTAGGGGAACTTCTCCTG
SARS-N1-B3	GTCCCCAAAATTTCCTTGG
SARS-N1-LF	AGCAAGAGCAGCATCACCG
SARS-N1-LB	CTGCCACTAAAGCATACAATGTAAC
<SARS-N2 set>	
SARS-N2-FIP	TCTGATTAGTTCCTGGTCCCCAAAGCATACAATGTAACACAAGC
SARS-N2-BIP	CGCATTGGCATGGAAGTCACTTTGATGGCACCTGTGTAG
SARS-N2-F3	GCAAAAACGTACTGCCAC
SARS-N2-B3	GAAATTTGGATCTTTGTCATCC
SARS-N2-LF	TGGACCACGTCTGCCGA
SARS-N2-LB	ACCTTCGGGAACGTGGTT

Table S4. Sequences of donor DNAs. Related to Fig. 2f, g, and S11.

Name	Sequence of homology arms (5′ to 3′)
mTyr-AAG-60b	GCATTACTATGTGTCAAGGGACACACTGCTTGGGGGCTCTGAAATATGGAGGGACATTGA
mTyr-AAG-Mut1-60b	GCATTACTATGTGTCAAGTGACACACTGCTTGGGGGCTCTGAAATATGGAGGGACATTGA
mTyr-AAG-Mut2-60b	GCATTACTATGTGTCAAGGTACACACTGCTTGGGGGCTCTGAAATATGGAGGGACATTGA
mTyr-AAG-Mut3-60b	GCATTACTATGTGTCAAGGGCCACACTGCTTGGGGGCTCTGAAATATGGAGGGACATTGA
mTyr-AAG-Mut4-60b	GCATTACTATGTGTCAAGGGAAACACTGCTTGGGGGCTCTGAAATATGGAGGGACATTGA
mTyr-AAG-Mut5-60b	GCATTACTATGTGTCAAGGGACCCACTGCTTGGGGGCTCTGAAATATGGAGGGACATTGA
mTyr-AAG-Mut6-60b	GCATTACTATGTGTCAAGGGACAAACTGCTTGGGGGCTCTGAAATATGGAGGGACATTGA
mTyr-AAG-Mut7-60b	GCATTACTATGTGTCAAGGGACACCCTGCTTGGGGGCTCTGAAATATGGAGGGACATTGA
mTyr-AAG-Mut8-60b	GCATTACTATGTGTCAAGGGACACAATGCTTGGGGGCTCTGAAATATGGAGGGACATTGA
mTyr-AAG-Mut9-60b	GCATTACTATGTGTCAAGGGACACACGGCTTGGGGGCTCTGAAATATGGAGGGACATTGA
mTyr-AAG-Mut10-60b	GCATTACTATGTGTCAAGGGACACACTTCTTGGGGGCTCTGAAATATGGAGGGACATTGA
mTyr-AAG-Mut11-60b	GCATTACTATGTGTCAAGGGACACACTGATTGGGGGCTCTGAAATATGGAGGGACATTGA
mTyr-AAG-Mut12-60b	GCATTACTATGTGTCAAGGGACACACTGCGTGGGGGCTCTGAAATATGGAGGGACATTGA
mTyr-AAG-Mut13-60b	GCATTACTATGTGTCAAGGGACACACTGCTGGGGGGCTCTGAAATATGGAGGGACATTGA
mTyr-AAG-Mut14-60b	GCATTACTATGTGTCAAGGGACACACTGCTTTGGGGCTCTGAAATATGGAGGGACATTGA
mTyr-AAG-Mut15-60b	GCATTACTATGTGTCAAGGGACACACTGCTTGTGGGCTCTGAAATATGGAGGGACATTGA
mTyr-AAG-Mut16-60b	GCATTACTATGTGTCAAGGGACACACTGCTTGGTGGCTCTGAAATATGGAGGGACATTGA
mTyr-AAG-Mut17-60b	GCATTACTATGTGTCAAGGGACACACTGCTTGGGTGCTCTGAAATATGGAGGGACATTGA
mTyr-AAG-Mut18-60b	GCATTACTATGTGTCAAGGGACACACTGCTTGGGGTCTCTGAAATATGGAGGGACATTGA
mTyr-AAG-Mut19-60b	GCATTACTATGTGTCAAGGGACACACTGCTTGGGGGATCTGAAATATGGAGGGACATTGA
mTyr-AAG-Mut20-60b	GCATTACTATGTGTCAAGGGACACACTGCTTGGGGGCGCTGAAATATGGAGGGACATTGA
mTyr-AAG-Mut21-60b	GCATTACTATGTGTCAAGGGACACACTGCTTGGGGGCTATGAAATATGGAGGGACATTGA
mTyr-AAG-Mut22-60b	GCATTACTATGTGTCAAGGGACACACTGCTTGGGGGCTCGGAAATATGGAGGGACATTGA
mTyr-AAG-Mut23-60b	GCATTACTATGTGTCAAGGGACACACTGCTTGGGGGCTCTTAAATATGGAGGGACATTGA
mTyr-AAG-Mut24-60b	GCATTACTATGTGTCAAGGGACACACTGCTTGGGGGCTCTGCAATATGGAGGGACATTGA
mTyr-AAG-Mut25-60b	GCATTACTATGTGTCAAGGGACACACTGCTTGGGGGCTCTGACATATGGAGGGACATTGA
mTyr-AAG-Mut26-60b	GCATTACTATGTGTCAAGGGACACACTGCTTGGGGGCTCTGAACTATGGAGGGACATTGA
mTyr-AAG-Mut27-60b	GCATTACTATGTGTCAAGGGACACACTGCTTGGGGGCTCTGAAAGATGGAGGGACATTGA
mTyr-AAG-Mut28-60b	GCATTACTATGTGTCAAGGGACACACTGCTTGGGGGCTCTGAAATCTGGAGGGACATTGA
mTyr-AAG-Mut29-60b	GCATTACTATGTGTCAAGGGACACACTGCTTGGGGGCTCTGAAATAGGGAGGGACATTGA
mTyr-AAG-Mut30-60b	GCATTACTATGTGTCAAGGGACACACTGCTTGGGGGCTCTGAAATATTGAGGGACATTGA
mTyr-AAG-Mut31-60b	GCATTACTATGTGTCAAGGGACACACTGCTTGGGGGCTCTGAAATATGTAGGGACATTGA
mTyr-AAG-Mut32-60b	GCATTACTATGTGTCAAGGGACACACTGCTTGGGGGCTCTGAAATATGGCGGGACATTGA
